# Comparison and assessment of family- and population-based genotype imputation methods in large pedigrees

**DOI:** 10.1101/gr.236315.118

**Published:** 2019-01

**Authors:** Ehsan Ullah, Raghvendra Mall, Mostafa M. Abbas, Khalid Kunji, Alejandro Q. Nato, Halima Bensmail, Ellen M. Wijsman, Mohamad Saad

**Affiliations:** 1 Qatar Computing Research Institute, Hamad Bin Khalifa University, Doha, Qatar;; 2 Division of Medical Genetics, Department of Medicine, University of Washington, Seattle, Washington 98195-9460, USA;; 3 Department of Biomedical Sciences, Joan C. Edwards School of Medicine, Marshall University, Huntington, West Virginia 25755, USA;; 4 Department of Biostatistics, University of Washington, Seattle, Washington 98195-9460, USA

## Abstract

Genotype imputation is widely used in genome-wide association studies to boost variant density, allowing increased power in association testing. Many studies currently include pedigree data due to increasing interest in rare variants coupled with the availability of appropriate analysis tools. The performance of population-based (subjects are unrelated) imputation methods is well established. However, the performance of family- and population-based imputation methods on family data has been subject to much less scrutiny. Here, we extensively compare several family- and population-based imputation methods on family data of large pedigrees with both European and African ancestry. Our comparison includes many widely used family- and population-based tools and another method, Ped_Pop, which combines family- and population-based imputation results. We also compare four subject selection strategies for full sequencing to serve as the reference panel for imputation: GIGI-Pick, ExomePicks, PRIMUS, and random selection. Moreover, we compare two imputation accuracy metrics: the Imputation Quality Score and Pearson's correlation *R*^2^ for predicting power of association analysis using imputation results. Our results show that (1) GIGI outperforms Merlin; (2) family-based imputation outperforms population-based imputation for rare variants but not for common ones; (3) combining family- and population-based imputation outperforms all imputation approaches for all minor allele frequencies; (4) GIGI-Pick gives the best selection strategy based on the *R*^2^ criterion; and (5) *R*^2^ is the best measure of imputation accuracy. Our study is the first to extensively evaluate the imputation performance of many available family- and population-based tools on the same family data and provides guidelines for future studies.

Genome-wide association studies (GWAS) have led to the discovery of hundreds of loci associated with complex diseases (Manolio et al. 2009; Visscher et al. 2017; Marigorta et al. 2018). Large sample sizes are required to achieve the necessary statistical power to identify such loci (Wang et al. 2005). Research consortia have attained large sample sizes by combining data from several studies using joint or meta-analysis (Evangelou et al. 2007; International Parkinson's Disease Genomics Consortium (IPDGC) and Wellcome Trust Case Control Consortium 2 (WTCCC2) 2011; International Parkinson Disease Genomics Consortium 2011; Siddiq et al. 2012; Nalls et al. 2014; Sniekers et al. 2017). These studies involved imputation of missing genotypes to allow association analysis of the same SNPs in multiple studies. Imputation facilitates performing joint or meta-analysis and also permits increasing the genomic coverage by searching for association on a much denser map. For all these reasons, performing imputation in GWAS data has become a common step (Marchini and Howie 2010). However, despite the large numbers of samples used, substantial heritability is not explained by the identified associations, leading to the conclusion that there is substantial rare variation that is also important and may explain part of the missing heritability (Maher 2008; Manolio et al. 2009; Visscher et al. 2017). Association with rare variants is difficult to find in analysis of unrelated subjects but can be identified in family-based designs, raising interest, once again, in family-based studies (Wijsman 2012).

To efficiently impute rare variants, imputation approaches that work well in general pedigrees are needed. To date, only a few methods have been proposed for family-based imputation designs, including Merlin (Burdick et al. 2006), GIGI (Cheung et al. 2013) coupled with gl_auto (Thompson 2011), PRIMAL (Livne et al. 2015), and cnF2freq (Nettelblad 2012). These approaches use, for example, sequencing data on a small set of subjects from the studied pedigrees and infer the missing genotypes on the remaining subjects (Saad and Wijsman 2014a). Unlike Merlin and GIGI, PRIMAL and cnF2freq are not set up for general use. Merlin and GIGI rely on identity by descent (IBD) computation, which is mostly identical for these tools and is based on the Lander-Green algorithm (Lander and Green 1987). The two main differences between the programs are their different approaches to the treatment of alleles in founders when such alleles are undefined by the data within the pedigree, together with their different capabilities for large pedigrees. GIGI can handle large pedigrees efficiently, whereas Merlin cannot, thus requiring pedigree splitting or trimming. In previous studies, the performance of GIGI was compared to several population-based imputation methods (Saad et al. 2016), while the performance of Merlin was separately evaluated on trimmed pedigrees (Lent et al. 2016). The two programs were not compared directly on large pedigrees, although other studies have shown that both GIGI and Merlin perform well for rare variant imputation but not as well for common variants (Chen et al. 2012; Saad and Wijsman 2014b). To date, there has not been an evaluation of all the approaches on the same data, used in a way that produces comparable results.

Population-based imputation coupled with phasing methods also exist. Phasing approaches include Eagle (Loh et al. 2016), MaCH (Li et al. 2010), IMPUTE (Howie et al. 2012), Beagle (Browning and Browning 2007), and SHAPEIT (Delaneau et al. 2012). Imputation approaches include minimac (Fuchsberger et al. 2015), IMPUTE (Howie et al. 2009), and Beagle (Browning and Browning 2016), and are more developed than family-based methods. Some of these methods have been compared in previous studies in both real and simulated data of unrelated subjects (Marchini and Howie 2010) and have also been extensively used in GWAS applications on real data of complex diseases (International Parkinson Disease Genomics Consortium 2011; Al-Tassan et al. 2015). The population-based imputation approaches can be used for imputation in family-based GWAS, but they ignore the IBD information and rely only on linkage disequilibrium (LD) information, which leads to a loss of information. They may lead to good imputation of common variants, but not for rare variants because of the minimal LD between rare variants (Saad and Wijsman 2014b). Moreover, in genomic regions where the LD is minimal between common variants, or the number of typed SNPs is low, IBD-based imputation methods may outperform population-based imputation methods for both rare and common variants. To benefit from both LD and IBD information, one can use the Ped_Pop (https://bioinformatics.qcri.org/ped_pop) approach (Saad and Wijsman 2014b), which combines family-based and population-based imputation methods using the best features of each to impute rare and common variants with higher accuracy. Although the original implementation of Ped_Pop combined GIGI and Beagle imputation results, the approach is general, and other combinations of methods could be used just as well. A comprehensive assessment of both family- and population-based imputation on pedigree data has not yet been done.

Imputation accuracy can be evaluated by several metrics. These include the concordance rate (CR), the imputation quality score (IQS) (Lin et al. 2010), and Pearson's squared correlation (*R*^2^). For common variants, these metrics provide similar accuracies, but for rare variants, this is not the case. For instance, the CR yields overestimated accuracy because common alleles are easily imputed (Lin et al. 2010). There is a need to know which accuracy metrics work well. Previous studies compared *R*^2^ and IQS (Ramnarine et al. 2015) but ignored the different meaning of these metrics, in that the *R*^2^ value is the squared correlation and the IQS is an agreement ratio. Moreover, the range of both metrics is not the same, with *R*^2^ varying from 0 to 1 while the upper bound of the IQS is one but the minimum could be negative. This precludes direct comparison of the *R*^2^ metric with IQS.

In imputation analysis, the selection of the reference data set has a great impact on the imputation accuracy. Unlike population-based imputation, which allows the use of external reference data sets, for example, the 1000 Genomes Project (The 1000 Genomes Project Consortium 2015), Haplotype Reference Consortium (HRC) (McCarthy et al. 2016), and UK10K (The UK10K Consortium 2015), family-based imputation requires the reference data set subjects to belong to the same pedigrees (Cheung et al. 2013; Saad and Wijsman 2014a). Several subject selection approaches exist for pedigree data: GIGI-Pick (Cheung et al. 2014), ExomePicks (http://genome.sph.umich.edu/wiki/ExomePicks), and PRIMUS (Staples et al. 2013). These approaches aim to select the pedigree members to be sequenced, forming the reference data set. GIGI-Pick capitalizes on the concept of inheritance vectors (IV) that represent the descent of chromosomes in a pedigree at specified positions. ExomePicks selects units of related subjects from the oldest to youngest generations, thus encouraging determination of haplotypes across loci. PRIMUS aims to identify a set of maximally unrelated subjects. The impact of these three approaches on the imputation accuracy of the different phasing and imputation approaches has not been thoroughly compared for both rare and common variants.

Here, we show the results of an extensive comparison of imputation methods in family-based data. Our data set consists of a collection of real pedigrees with small to large sizes. We simulated genetic data on these pedigrees to mimic the minor allele frequency spectrum and LD of the 1000 Genomes Project to evaluate results for both European and African ancestries. We compared the main family- and population-based combinations of phasing and imputation algorithms: gl_auto, GIGI, Merlin, Eagle, SHAPEIT (with and without the duoHMM feature), MaCH, minimac, IMPUTE, Beagle, and Ped_Pop. To run Merlin, we split all pedigrees into smaller subpedigrees that can fit in the memory and then combined the subpedigree results. We also compared the effect of four subject selection strategies—GIGI-Pick, ExomePicks, PRIMUS, and random selection—on the imputation accuracy. Finally, we compared the imputation accuracy measures *R*^2^ and IQS for various minor allele frequency (MAF) intervals with respect to the power of association analysis using a linear mixed model. We ignored the concordance rate because of the well-known limitation mentioned earlier (Ramnarine et al. 2015). Our paper represents the first comprehensive guideline to the choice of imputation methods in family-based human genetic data, and delivers answers regarding the choice of the best subject selection for downstream association analysis, and which phasing and imputation methods to use, depending on the context and scenarios of a study.

## Results

### Mean squared correlation (*R*^2^)

**Figure 1.**
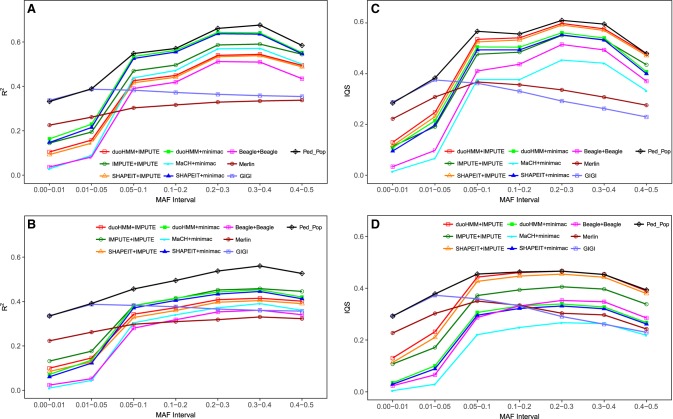
Mean correlation *R*^2^ and IQS between true and imputed genotypes for all approaches, using the random selection strategy: (*A*) *R*^2^ for EUR; (*B*) *R*^2^ for AFR; (*C*) IQS for EUR; (*D*) IQS for AFR. The first/second of a pair of programs in the key indicates phasing/imputation functions. Computation of the mean of *R*^2^ and IQS is based on all 100 genetic data sets with a sample size of 960 subjects, each having 7954 SNPs for EUR and 10,891 SNPs for AFR.

The *R*^2^ values were estimated between the imputed and true observed dosages. Here, the dosage is the estimated (or observed) fraction of minor alleles in the genotype. This computation was performed for all SNPs except the observed GWAS SNPs (i.e., 500 in both EUR and AFR), which were not imputed. The results of mean *R*^2^ for random selection are summarized in Figure 1, A and B. This figure shows that for rare or infrequent variants (MAF in [0,0.05]), family-based imputation methods outperformed population-based methods. GIGI had the same performance in both European (EUR) and African (AFR). The same trend was also observed for Merlin. Within family-based approaches, GIGI (using full pedigrees) outperforms Merlin (using subpedigrees) across all MAF intervals for both EUR and AFR. When applied on the same subpedigrees, GIGI outperformed Merlin for the rare variants but not for the common ones (Supplemental Fig. S1). Zooming in on the (0,0.05) MAF interval, Supplemental Figure S2 shows how the different methods behave for rare variants and how the clear improvement of population-based approaches starts to be apparent between [0.03,0.04) and [0.04,0.05).

Imputation of the more common variants was better with population-based than pedigree-based methods (MAF in [0.05,0.5]) (Fig. 1A,B). The better performance for population-based imputation is more evident in the EUR compared to the AFR sample, for which GIGI and Merlin were not substantially outperformed for the common variants. Within population-based approaches, duoHMM for phasing followed by minimac for imputation (duoHMM+minimac) and SHAPEIT+minimac were the best combinations for the EUR across all MAF intervals (Fig. 1A). In the AFR, IMPUTE+IMPUTE performed as well as those two previous combinations (Fig. 1B). To check if the differences between population-based approaches were simply due to sampling variation, we regenerated 100 new genetic data sets using the random selection strategy, and we performed imputation combinations MaCH+minimac, SHAPEIT+minimac, duoHMM+minimac, IMPUTE+IMPUTE, SHAPEIT+IMPUTE, and duoHMM+IMPUTE for EUR and AFR. The same differences and trends were observed again, which suggests that these difference are systematic (Supplemental Fig. S3). Note that Beagle had the lowest imputation accuracy. Moreover, for all population-based methods, our results showed that imputation accuracy was greater for EUR than AFR (Fig. 1A versus Fig. 1B). Notably, the hybrid approach Ped_Pop, which combined both family- and population-based strengths, had the greatest performance for EUR and AFR in case of both rare and common variants (Fig. 1). Supplemental Figure S4 shows an example of GIGI, duoHMM+minimac, and Ped_Pop performances for imputing two arbitrary chosen SNPs (one with MAF = 0.006 and one with MAF = 0.46) in a pedigree of 24 subjects, of whom five subjects were fully observed. For the SNP with low allele frequency (MAF = 0.006), GIGI perfectly imputed all 10 genotypes with at least one copy of the minor allele, whereas duoHMM+minimac could not impute any of them. For the SNP with high allele frequency (MAF = 0.46), duoHMM+minimac accurately imputed all 14 genotypes with at least one copy of the minor allele, whereas GIGI could impute 11. In both cases, Ped_Pop accurately imputed genotypes with at least one copy of the minor allele.

### Eagle vs. SHAPEIT for phasing

**Figure 2.**
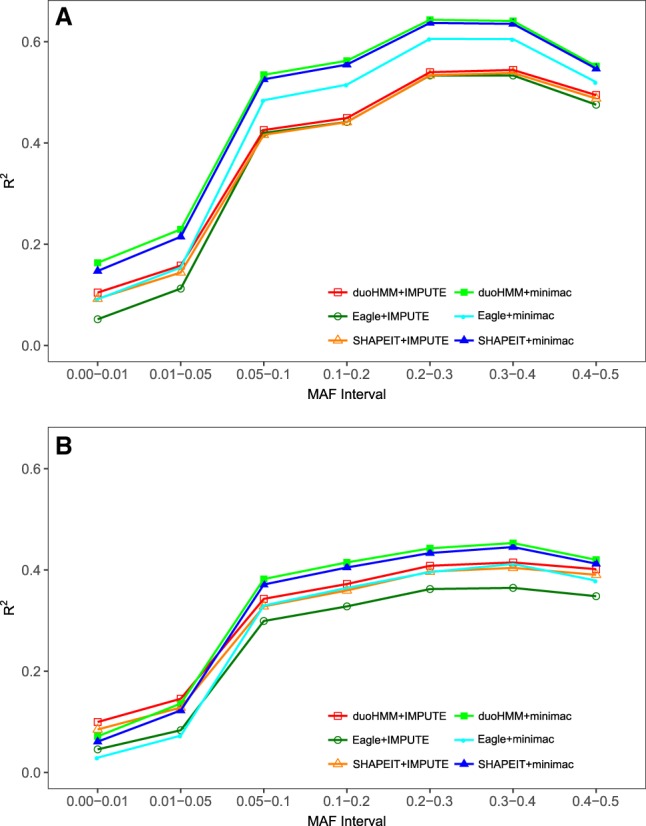
Mean correlation *R*^2^ between true and imputed genotypes for SHAPEIT+minimac, duoHMM+minimac, SHAPEIT+IMPUTE, duoHMM+IMPUTE, Eagle+minimac, and Eagle+IMPUTE, using the random selection strategy: (*A*) EUR; (*B*) AFR. The first/second of a pair of programs indicates phasing/imputation functions. Computation of the mean of *R*^2^ is based on all 100 genetic data sets with a sample size of 960 subjects, each having 7954 SNPs for EUR and 10,891 SNPs for AFR.

Figure 2, A and B, show that the use of SHAPEIT or duoHMM for phasing was more successful at yielding high-quality imputed data than the use of Eagle. When phasing was done with Eagle, imputation accuracy dropped, especially with minimac. This pattern was apparent for both the EUR (Fig. 2A) and AFR (Fig. 2B) data and for all MAF intervals.

### IQS vs. *R*^2^ and statistical power as a baseline

IQS and *R*^2^ were not always in agreement in summarizing imputation accuracy (Fig. 1A,B versus Fig. 1C,D, respectively). Similar conclusions reached using either IQS or *R*^2^ were that (1) Ped_Pop performed best overall; (2) family-based approaches had higher values for rare variants, whereas population-based approaches had higher values for common variants; and finally, (3) both IQS and *R*^2^ yielded a better performance in the EUR data compared to AFR data. IQS differed from *R*^2^, with (1) the approaches involving IMPUTE for imputation were better than the other approaches for both EUR and AFR (Fig. 1C versus Fig. 1D); (2) the approaches involving minimac for imputation appeared to be less accurate; (3) MaCH+minimac, and not Beagle, appeared to be the worst approach; and finally, (4) GIGI was slightly outperformed by Merlin for common variants. Altogether, the IQS values were smaller than the *R*^2^ values for all approaches as can be seen, for instance, for GIGI and duoHMM+minimac in Supplemental Figure S5. This does not necessarily mean that *R*^2^ values overestimate imputation accuracy as claimed by Ramnarine et al. (2015).

To determine the metric that appears to be better for imputation accuracy, we computed the statistical power of association analysis and used this as a baseline to identify the imputation approach that leads to the highest statistical power. We focused on the cases in which a disagreement between IQS and *R*^2^ was observed. For these cases, we compared the corresponding powers. Type 1 error rates were well controlled for all observed genotype and imputed dosages for the Random and GIGI-Pick selection strategies for *α* = 0.05, 0.01, and 0.001 with the exception of evidence for slight conservatism for the population-based imputation programs in the bin with the lowest MAF (Supplemental Tables S1–S6). Power results for the random selection strategy for *α* = 0.05 are shown in Table 1, and the remaining tables are shown in Supplemental Tables S7–S11.

**Table 1.**
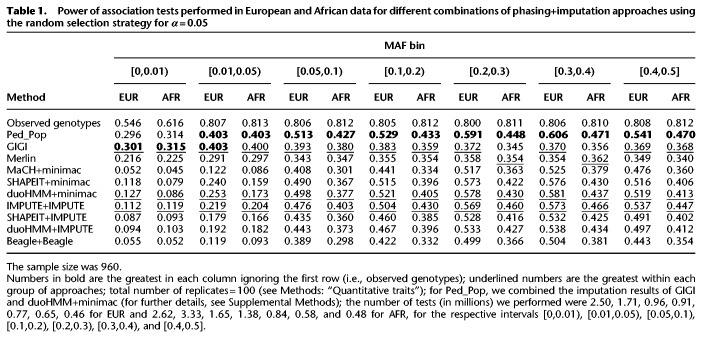
Power of association tests performed in European and African data for different combinations of phasing+imputation approaches using the random selection strategy for ***α*** = 0.05

The power results showed that SHAPEIT+minimac and duoHMM+minimac were better than SHAPEIT+IMPUTE and duoHMM+IMPUTE. They also showed that the power of Beagle was slightly lower than MaCH+minimac. Finally, the power results showed that GIGI had at least similar power compared to Merlin. All of these results are in agreement with the *R*^2^ values. In addition, these conclusions were most obvious for common variants. For rare variants, the trend was similar despite the small values of both IQS and *R*^2^.

In the association analysis, the direct power estimates for Ped_Pop were the largest among all the imputation approaches (Table 1). Power to detect association was smaller in AFR than EUR for all population-based imputation. On the other hand, power achieved through the use of GIGI for imputation was similar in EUR and AFR. Finally, power for imputation involving the GIGI-Pick selection was higher than for the random selection, for all *α* levels examined (Table 1 versus Supplemental Table S9; Supplemental Table S7 versus Supplemental Table S10; and Supplemental Table S8 versus Supplemental Table S11). Again, all these results are congruent with the imputation *R*^2^ accuracy values.

### Subject selection strategies

**Figure 3.**
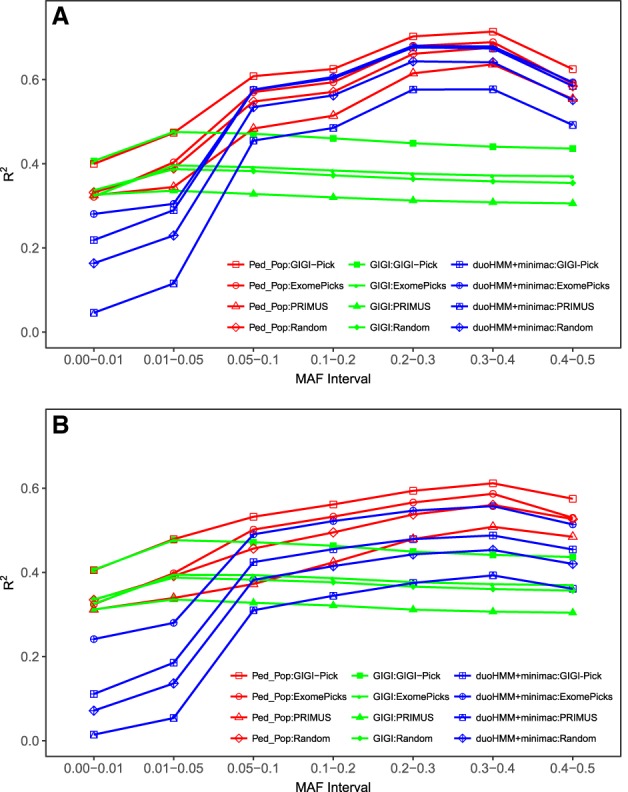
Mean correlation *R*^2^ between true and imputed genotypes for the four selection strategies (GIGI-Pick, ExomePicks, PRIMUS, and random selection) for Ped_Pop, GIGI, and duoHMM+minimac: (*A*) EUR; (*B*) AFR. Computation of the mean of *R*^2^ is based on all 100 genetic data sets with a sample size of 960 subjects, each having 7954 SNPs for EUR and 10,891 SNPs for AFR.

The four subject selection strategies we compared performed differently depending on the phasing+imputation combination used. In Figure 3, A and B, we show the mean *R*^2^ for three imputation approaches in EUR and AFR: GIGI (best of family-based imputation), duoHMM+minimac (best of population-based imputation), and Ped_Pop (combination of GIGI and duoHMM+minimac). As expected, PRIMUS did not perform well for any imputation method; it was even worse than the random selection strategy. The reason behind this result is that in family data, subjects with vertical and horizontal relationships in pedigrees (e.g., parent-offspring, siblings) improve the phasing process and therefore the imputation. By repeating a random selection on all simulated data sets, such relationships were present more often than with PRIMUS, which forces the selection of a set of maximally unrelated subjects.

The selection of subjects using GIGI-Pick led to better imputation accuracy than ExomePicks for both GIGI and Ped_Pop using the *R*^2^ criterion. Note that when imputing rare variants, ExomePicks selection led to a better imputation accuracy than GIGI-Pick for SHAPEIT+minimac, SHAPEIT+IMPUTE, MaCH+minimac, duoHMM+minimac, duoHMM+IMPUTE, and Beagle (Supplemental Fig. S6A). However, imputation accuracies of these approaches remained much smaller than GIGI's accuracy. For common variants, the same trend was observed, but with smaller differences between GIGI-Pick and ExomePicks (Supplemental Fig. S6B). All the preceding conclusions were the same for AFR.

## Discussion

Our study is the first to address several challenges faced in imputation in family data and evaluate the performance of many available family- and population-based tools in GWAS analysis on the same family data of both European and African ancestry, and it provides guidelines for future studies. We showed that family-based imputation outperforms population-based imputation for rare variants. For common variants, population-based approaches are expected to be better except when the amount of LD between SNPs is low. This explains why population-based imputation yielded more accurate results on data from European than African samples (mean LD computed within nonoverlapping windows of 100 SNPs in EUR was *R*^2^ = 0.032 versus 0.02 in AFR). It is worth noting, however, that family-based imputation was not affected by the ancestry differences because this approach relies on IBD rather than LD.

Of the population-based tools, the combination of SHAPEIT (v2) with the duoHMM feature for phasing and minimac (v3) for imputation outperformed all other combinations. For family-based imputation, we found that GIGI outperformed Merlin for rare variants but not for the common ones. This is because Merlin uses LD information by incorporating the fastPHASE algorithm (Scheet and Stephens 2006) when IBD information cannot determine the phase. But because population-based approaches outperformed both GIGI and Merlin for common variants, GIGI would be preferable to Merlin from both an accuracy and computational point of view. Merlin presented great computational challenges even for small sets of SNPs (<11,000 SNPs) and also required splitting pedigrees into smaller subpedigrees. Therefore, running Merlin on a GWAS or Whole-Genome Sequence level would be impractical. The solution that worked best across the full range of allele frequencies was that implemented in Ped_Pop, which combines the strengths of both family- and population-based imputation. By considering both EUR and AFR populations, we combined the results of GIGI and duoHMM+minimac (for further details, see Supplemental Methods). This approach led to the greatest imputation accuracy and largest association power for both rare and common variants.

The accuracy measure used to evaluate the imputation performance is of great importance. The accuracy results computed with *R*^2^ were concordant with the power of association analysis, contrary to the results computed with IQS, which provided an inconsistent predictor of statistical power. Overall, our results suggest that IQS underestimates imputation accuracy, but *R*^2^ better defines the imputation accuracy and should continue to be used to evaluate imputation accuracy for both rare and common variants in future studies. In addition, *R*^2^ has a direct relationship with power in association studies. For example, in the case of imputing one SNP on 1000 unrelated subjects, an imputation accuracy of *R*^2^= 0.8 means that to achieve the same power when using perfect genotypes, the sample size must be 1/0.8 = 1.25 times higher.

The choice of subjects to be sequenced from pedigrees has a large impact on the imputation performance and therefore on the association results. A careful and optimal choice of selected subjects at the study design level would likely increase the imputation accuracy and therefore the power of association tests. In our study, we evaluated four strategies to select subjects for sequencing and analysis with an association test on the imputation accuracy: random selection strategy, GIGI-Pick, ExomePicks, and PRIMUS. Our results showed that if one is interested in rare variant imputation, the selection of subjects should be done using GIGI-Pick and imputation should be done using GIGI. If one is interested in both rare and common variant imputation, the selection of subjects and imputation should be done using GIGI-Pick and Ped_Pop, respectively.

Here, we did not evaluate pedigrees chosen through selective phenotypic ascertainment, but we believe that our general conclusions should still hold under such ascertainment. Pedigree ascertainment through selective phenotypes can only affect power to detect true associations. Power is a function of the number of subjects with high-quality imputed genotypes, particularly in individuals who are not closely related. Of the available tools for subject selection, GIGI-Pick, with its genome-wide option used here, already does the best job of balancing the conflicting needs of resequencing inherited copies of genomic regions for phasing, while also distributing the sequencing across individuals without shared inheritance. More importantly, GIGI-Pick is also currently the only subject selection tool that can also take into account realized IBD in a region of interest for subject selection. Such a region may be determined with pedigrees selected through members’ phenotypic status followed by genotyping with a low-cost SNP array and linkage analysis. As was shown previously, this option can have a marked positive effect on the overall number of high-quality, imputed, relatively independent genotypes in the region (Cheung et al. 2014), and thus the power of an association test.

In our study, we did not evaluate the performance of phasing approaches but only compared the imputation accuracy with respect to the different phasing methods used. Two of the best population-based phasing algorithms are Eagle and SHAPEIT. In our simulated data, we observed that SHAPEIT outperformed Eagle, which was also observed in Herzig et al. (2018), contrary to what was observed by Eagle's authors (Loh et al. 2016). For family-based phasing, we used gl_auto to phase the set of sparse markers. Like GIGI, gl_auto does not use LD for phasing and imputation. Future incorporation of such useful information into GIGI will most certainly improve its performance, and may, ultimately, outperform the Ped_Pop approach. Until this happens, the results of our study suggest that the approach of Ped_Pop (https://bioinformatics.qcri.org/ped_pop) provides a pragmatic approach that combines both pedigree- and population-based strengths.

## Methods

### Simulated data

#### Genetic data

We simulated sequence data on a collection of 20 extended pedigrees consisting of 1200 total subjects with sizes ranging from 10 to 174 subjects and with median and mean sizes of 47 and 60 subjects, respectively. The sibship sizes ranged from 1 to 11 siblings, with median and mean sizes of 1 and 1.86 sibling, respectively. The number of generations ranged from 3 to 9, with median and mean sizes of 8 and 6.65 generations, respectively. The pedigree, generation, and sibship sizes were modeled on those of real pedigrees (EM Wijsman, pers. comm.). The generated pedigrees and the simulated sequenced data are available online (Data Access). We used the same simulation strategy used in a previous study (Saad and Wijsman 2014a) to obtain 100 semirealistic sequence data sets that mimic the LD structure and MAF spectrum of the 1000 Genomes Project for European and African ancestries (called EUR and AFR throughout). Briefly, for each ancestry, we simulated 20,000 haplotypes for a region of ∼6 Mb pairs on Chromosome 22 (Genome built GRCh37, 26443384–32049917) using HAPGEN (Su et al. 2011). From the pool of 20,000 haplotypes, we started by randomly selecting haplotypes, without replacement, for the unrelated founders. Then, we dropped the haplotypes from the founders down through the 3–5 generations in pedigrees using a recombination rate of 1% per centiMorgan (cM) per meiosis under the assumption that 1 cM is 1000 kb pairs. We used the same pedigrees for both EUR and AFR. The number of SNPs in the EUR and AFR 1000 Genomes data were 8954 and 11,891, respectively. For both EUR and AFR, 500 SNPs (∼5% of the total number of SNPs) were randomly selected to form the GWAS list of SNPs. The whole process was repeated 100 times to finally obtain 100 simulated data sets for the two ancestries. The distributions of MAFs for both EUR and AFR are shown in Supplemental Figure S7. Note that reference assembly for sequence read alignment (e.g., GRCh37 or GRCh38) has no significant impact on the linkage disequilibrium, or IBD. Therefore, our conclusions will not be affected by the use of the GRCh37 reference assembly.

#### Quantitative traits

We simulated quantitative traits by sampling from two models: (*H*_0_) *Y* = *ε*, (*H*_*a*_) *Y* = *β*_*j*_*X*_*j*_ + *ε*. In both models, ε follows a multivariate normal distribution *N*(0, Σ) where Σ = *h*^2^Φ + (1 − *h*^2^)*I*, Φ is the matrix of twice the kinship coefficient between pairs of subjects, *I* is the identity matrix, and *h*^2^ is the heritability, set to 0.5 by setting the total variance to 2 and the genetic variance due to polygenic effects to 1. In model *H*_*a*_, *X*_*j*_ is the variable of known genotypes of the *j*th SNP coded as 0, 1, or 2 copies of the minor alleles, *β*_*j*_ is the effect size of the corresponding SNP and calculated as

$$\beta_{j}=\sqrt{\frac{v_{a}}{2\times \mathrm{MA}F_{j}\times (1-\mathrm{MA}F_{j})}}\;\;,$$

where MAF_*j*_ is the minor allele frequency and *v*_*a*_, set to 0.01, is the additive variance of SNP *j*.

For each SNP and each genetic data set, we simulated 10 quantitative traits for the *H*_0_ model (hypothesis of no association) to compute the type 1 error rate and 10 quantitative traits for the *H*_*a*_ model (hypothesis of association) to compute the statistical power rate. Because there are 100 genetic data sets and 10 quantitative traits for each data set, we calculated the rate for each SNP as a proportion of these 1000 data sets. We computed both rates for each SNP as the proportion of replicates with a *P*-value smaller than a given *α* and then averaged all SNP rates within the following MAF bins: (0,0.01), [0.01,0.05), [0.05,0.1), [0.1,0.2), [0.2,0.3), [0.3,0.4), and [0.4,0.5]. This process yielded the following number of tests within the respective MAF bins: 2,497,900, 1,708,680, 955,460, 911,850, 766,810, 652,040, 461,260 for EUR and 2,621,320, 3,333,930, 1,646,340, 1,384,900, 840,620, 582,290, and 481,600 for AFR. We used three values of *α*: 0.05, 0.01, and 0.001. Note that more stringent thresholds could be applied, which will likely yield lower power. For the sake of comparing the different imputation approaches and not evaluating the statistical power per se, the *α* thresholds we used would be enough to reach our main conclusions.

### Imputation and association analyses

In all approaches evaluated, imputation relies on inferring missing genotypes in study subjects using a reference data set of fully sequenced subjects. The study subjects are genotyped on a sparse map of SNPs. All imputation methods are based on two steps: phasing and imputation. For family-based imputation, the reference data set only needs to contain subjects from the pedigrees under study. For population-based imputation methods on pedigree data, the reference data set was formed by combining all sequenced subjects across pedigrees. In all the imputation analyses, we selected subjects based on the pedigree structure from which we simulated sequence data in order to obtain the reference data set. In all tools we compared, the default parameters suggested by their respective developers were used.

#### Selection of reference data set

An optimal selection choice, in the context of trait mapping, depends on several factors: the pedigree structure, the availability of phenotype, the severity of disease, and the availability of good-quality DNA. In our simulation study, we only used the pedigree structure to select subjects. We compared four selection strategies: GIGI-Pick (Cheung et al. 2014), ExomePicks (http://genome.sph.umich.edu/wiki/ExomePicks), PRIMUS (Staples et al. 2013, 2014), and random selection. In all imputation analyses, 20% of subjects were selected for sequencing from each of the 20 analyzed pedigrees. This proportion was used across all pedigrees, resulting in 240 subjects. For all four selection strategies, the set of 240 subjects obtained was used for EUR and AFR imputation because the pedigree structure was the same. A detailed description about the subject selection procedures is provided in the Supplemental Material.

The proposed phasing and imputation algorithms that were assessed in our study are listed next, and we briefly summarize them in Table 2. A more thorough description can be found in the Supplemental Material.

**Table 2.**
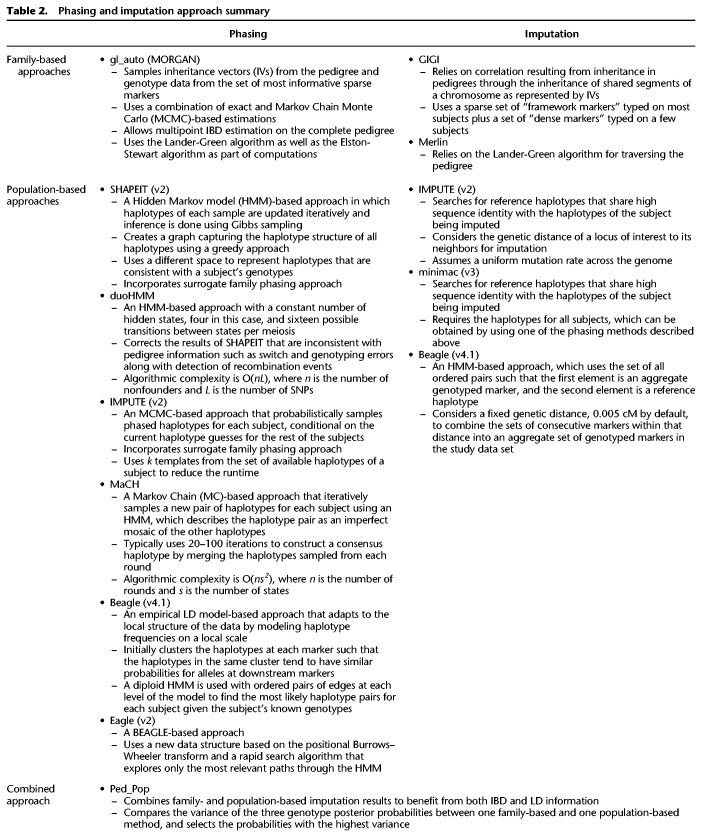
Phasing and imputation approach summary

### Family-based design imputation

We used pedigree-based imputation computer programs GIGI (Cheung et al. 2013) and Merlin (Abecasis et al. 2002). GIGI requires a prephasing step in which the IBD is computed by the program gl_auto, implemented in MORGAN (Thompson 2011). On the other hand, Merlin performs this step internally. Running Merlin on large pedigrees requires too much memory, which can be predicted by the number of bits in the pedigree (Kruglyak et al. 1996), bits = 2*n* − *f*, where *n* is the number of nonfounders, and *f* is the number of founders. The pedigrees we are using ranged from 5 to 165 bits. In our data, imputation of pedigrees with 19 bits required 110 GB of memory to impute 8954 SNPs. To get results from Merlin, we split large pedigrees into small computable subpedigrees defined with a maximum of 19 bits.

Automated methods for subdividing the pedigree structures exist, such as PedCut (Liu et al. 2008) and PedStr (Kirichenko et al. 2009). However, we found them unsatisfactory, resulting in excessively small subpedigrees without the flexibility to ensure that at least some sequenced subjects are in each subpedigree. We instead opted to manually construct the subpedigrees to include a greater number of subjects in each subpedigree and to be close to the upper limit of 19 bits, while including both vertical and horizontal relationships (grandparents, parents, offspring, siblings, etc.). We often included the same individuals in several of the subpedigrees to be closer to 19 bits. In these cases, we retained the imputation results for these individuals from the largest subpedigree when combining the results.

### Population-based design imputation

For phasing, we used the following programs: SHAPEIT (v2) (Delaneau et al. 2012), duoHMM (O'Connell et al. 2014), IMPUTE (Howie et al. 2009), MaCH (Li et al. 2010), Beagle (Browning and Browning 2007), and Eagle (Loh et al. 2016). For imputation, we used: IMPUTE, minimac (v3) (Fuchsberger et al. 2015), and Beagle (v4.1) (Browning and Browning 2016). The versions of the tools we used are the latest only from an algorithmic point of view. For example, we used IMPUTE (v2), which has a newer version (v4; https://jmarchini.org/impute-4/). However, IMPUTE phasing and imputation algorithms did not change in the new version. The new phasing and imputation versions that are being proposed are mainly aiming at handling larger data sets.

### Combination of family- and population-based imputation

To benefit from both IBD and LD information, we combined family- and population-based imputation results using Ped_Pop (https://bioinformatics.qcri.org/ped_pop) (Saad and Wijsman 2014b). Ped_Pop can combine imputation results from any family- and population-based methods. In this study, the family- and population-based approaches with overall best performance were combined (for further details, see Supplemental Methods).

### Imputation accuracy measures

Several measures to compute imputation accuracy have been proposed. Examples include Pearson's correlation *R*^2^, the imputation quality score (IQS) (Lin et al. 2010) based on the Kappa statistic, and the concordance rate (CR). The performance of these metrics depends on the MAF of imputed SNPs. For example, CR overestimates the imputation accuracy for rare variants. Moreover, it also has been claimed that *R*^2^ overestimates imputation accuracy for rare variants (Lin et al. 2010). However, the claim was based on comparing *R*^2^ and IQS values, without reference to a baseline to decide which metric is better, while knowing that these values cannot be compared directly. Here, we compared *R*^2^ and IQS and their behavior with respect to the statistical power of the association test to determine the best metric. The imputation that leads to the highest association power is the best imputation approach in this context. The use of type 1 error rates does not allow comparison of *R*^2^ and IQS because they are expected to be similar and close to the *α* threshold we set (e.g., *α* = 0.05) in all scenarios. Note that we chose not to use the CR in our comparison because of its known limitations.

### Association testing

We constructed the following linear mixed model to test for association between SNPs and quantitative traits: *Y* = *β*_*j*_*X*_*j*_ + *ε* where *X*_*j*_ is the variable representing the genotype of the *j*th SNP coded as the number of copies of the minor alleles, *β*_*j*_ is the corresponding regression coefficient, and $\epsilon ∼N(\beta_{j}X_{j},\sigma_{g}^{2}\Phi +\sigma_{e}^{2}I)$ where Φ is the matrix of twice the kinship coefficient between pairs of subjects, $\sigma_{e}^{2}$ is the residual variance, and $\sigma_{g}^{2}$ is the polygenic variance. Association tests were performed twice (under the null and alternative hypotheses) on all SNPs except the first and the last 500 SNPs, where imputation results for population-based imputation are poor due to lack of buffer downstream and upstream. The association test was performed on the data of true genotypes and also on the data of all imputation combinations without applying any poor-quality imputation filtering. The number of tests we performed were 2,497,900, 1,708,680, 955,460, 911,850, 766,810, 652,040, 461,260 for EUR and 2,621,320, 3,333,930, 1,646,340, 1,384,900, 840,620, 582,290, and 481,600 for AFR for the respective intervals [0,0.01), [0.01,0.05), [0.05,0.1), [0.1,0.2), [0.2,0.3), [0.3,0.4), and [0.4,0.5]. These analyses were conducted for the observed genotype dosages and the imputed ones for the Random and GIGI-Pick selection strategies using the “lmekin” function in the “kinship2” R package (https://cran.r-project.org/web/packages/kinship2/index.html).

Finally, in our study, we evaluated several combinations of phasing and imputation methods in family- and population-based designs in both EUR and AFR. These combinations (Phasing+Imputation) were the following: MaCH+minimac, SHAPEIT+minimac, duoHMM+minimac, Eagle+minimac, IMPUTE+IMPUTE, SHAPEIT+IMPUTE, duoHMM+IMPUTE, Eagle+IMPUTE, Beagle+Beagle, GIGI (using gl_auto), Merlin, and Ped_Pop (combining GIGI and duoHMM+minimac). All approaches were performed for the four selection strategies: GIGI-Pick, ExomePicks, PRIMUS, and random selection. Note that Merlin was run for the subpedigrees only with the GIGI-Pick and random selection strategies. To have a fair comparison with GIGI, GIGI was run on the same subpedigrees and also on the full, large pedigrees.

## Data access

Simulated genetic sequence data and pedigree structures used in our study are available at Zenodo with DOI: 10.5281/zenodo. 1485558 (https://zenodo.org/record/1485558) and on our site at https://bioinformatics.qcri.org/IRD.

## Supplementary Material

Supplemental Material
