# Supplementary material for: Comparison and assessment of family- and population-based genotype imputation methods in large pedigrees
Source: Genome Res. 2019 Jan;29(1):125–34. doi: 10.1101/gr.236315.118 (PMC6314157; doi:10.1101/gr.236315.118)
Supplement: Supplemental Material [file supp_29_1_125__index.html]

Comparison and assessment of family- and population-based genotype imputation methods in large pedigrees — Comparison and assessment of family- and population-based genotype imputation methods in large pedigrees — Supplemental Material 

# Comparison and assessment of family- and population-based genotype imputation methods in large pedigrees

## Supplemental Material

- Supplemental\_Fig\_S1.pdf
- Supplemental\_Fig\_S2.pdf
- Supplemental\_Fig\_S3.pdf
- Supplemental\_Fig\_S4.pdf
- Supplemental\_Fig\_S5.pdf
- Supplemental\_Fig\_S6.pdf
- Supplemental\_Fig\_S7.pdf
- Supplemental\_Table\_S1.xlsx
- Supplemental\_Table\_S2.xlsx
- Supplemental\_Table\_S3.xlsx
- Supplemental\_Table\_S4.xlsx
- Supplemental\_Table\_S5.xlsx
- Supplemental\_Table\_S6.xlsx
- Supplemental\_Table\_S7.xlsx
- Supplemental\_Table\_S8.xlsx
- Supplemental\_Table\_S9.xlsx
- Supplemental\_Table\_S10.xlsx
- Supplemental\_Table\_S11.xlsx
- Supplemental\_Methods.docx
