# Supplementary material for: Comparison and assessment of family- and population-based genotype imputation methods in large pedigrees
Source: Genome Res. 2019 Jan;29(1):125–34. doi: 10.1101/gr.236315.118 (PMC6314157; doi:10.1101/gr.236315.118)
Supplement: Supplemental Material [file supp_gr.236315.118_Supplemental_Fig_S1.pdf]

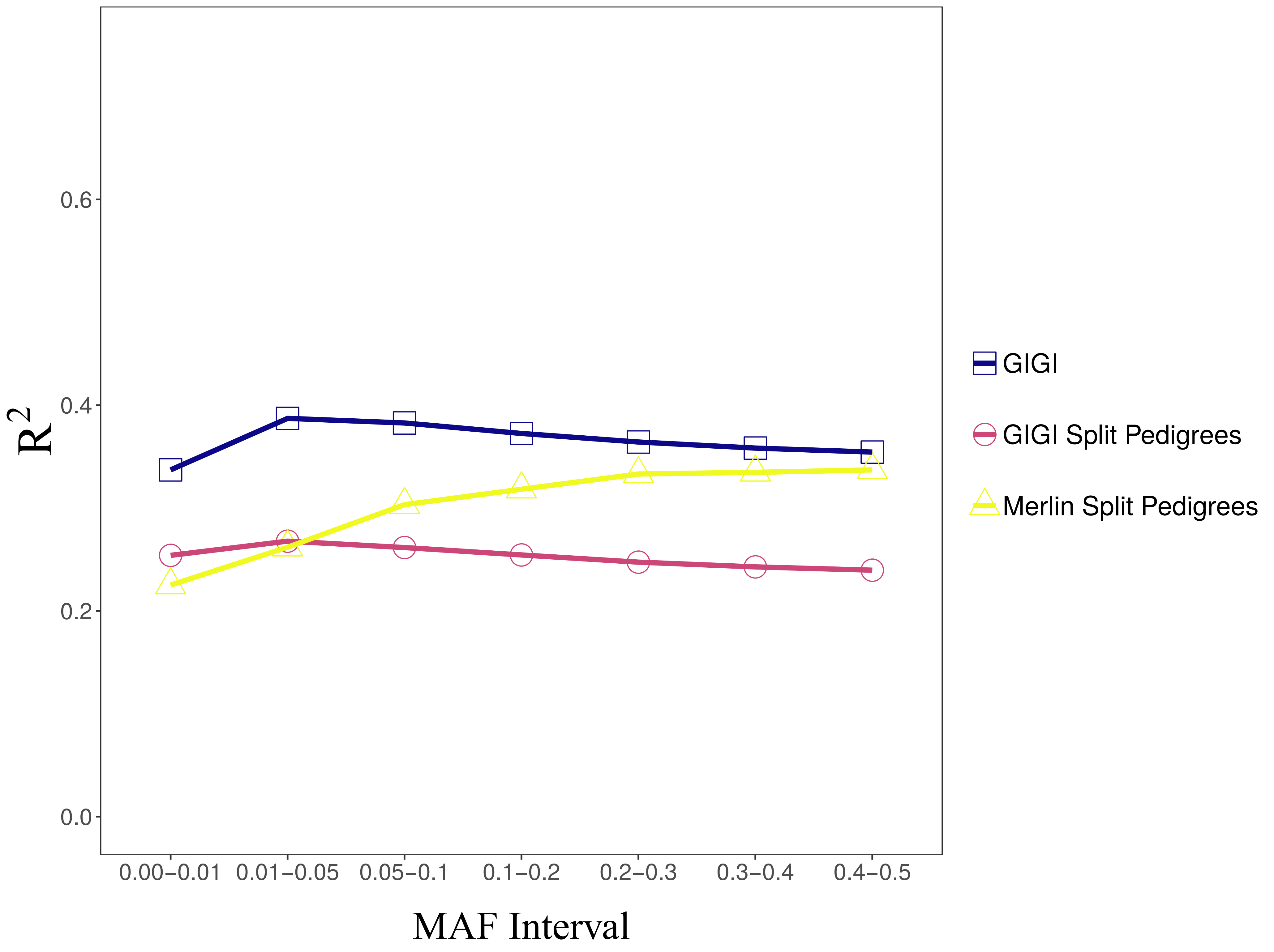

Supplemental Figure S1: Mean correlation R^2 between true and imputed genotypes for GIGI and Merlin using sub-pedigrees, and also GIGI using whole pedigrees without splitting in EUR using the random selection strategy.
