# Supplementary material for: Comparison and assessment of family- and population-based genotype imputation methods in large pedigrees
Source: Genome Res. 2019 Jan;29(1):125–34. doi: 10.1101/gr.236315.118 (PMC6314157; doi:10.1101/gr.236315.118)
Supplement: Supplemental Material [file supp_gr.236315.118_Supplemental_Fig_S2.pdf]

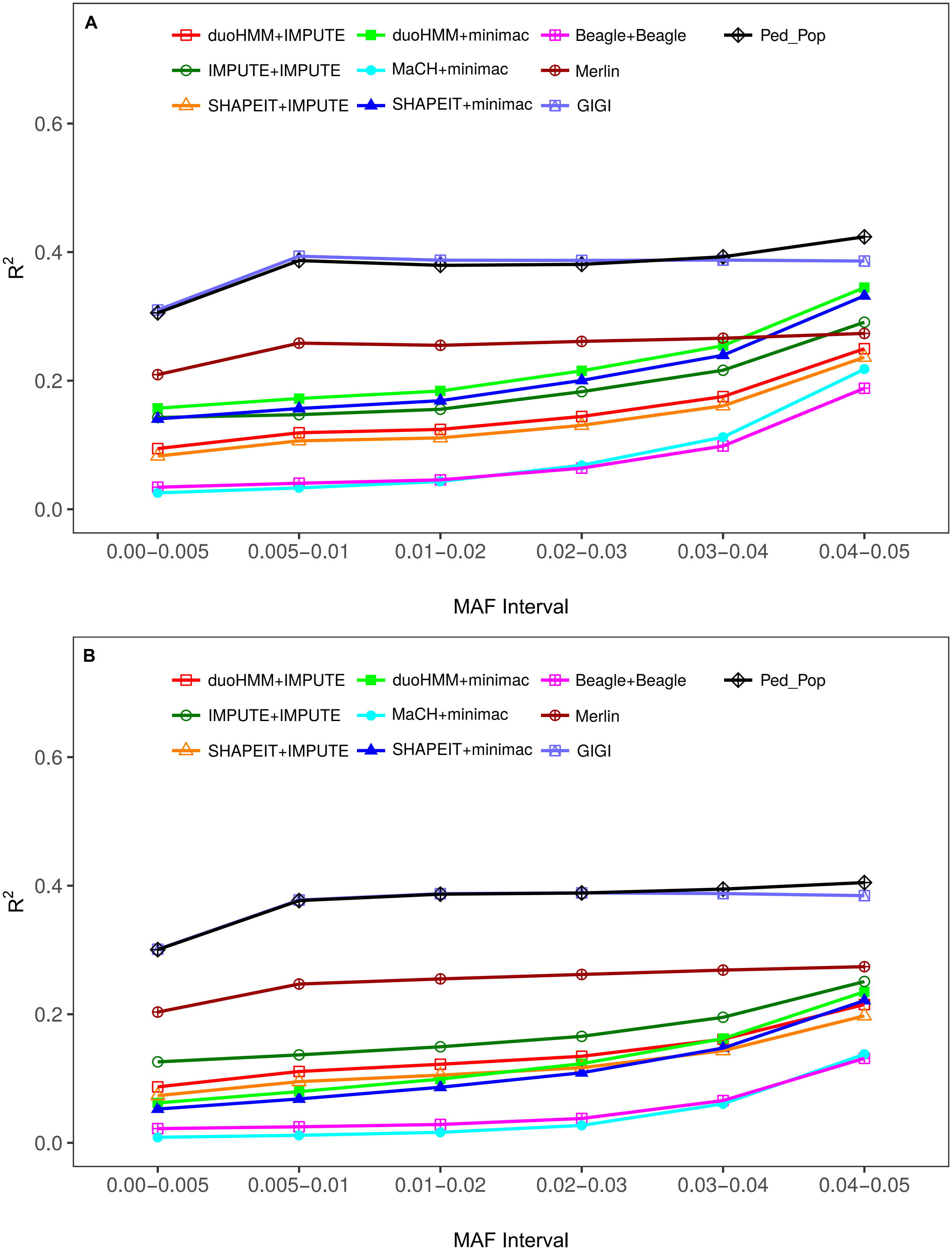

Supplemental Figure S2: Mean correlation R^2 between true and imputed genotypes for all approaches with a zoom in the MAF interval [0,0.05) for (A) EUR and (B) AFR using the random selection strategy.The first/second of a pair of programs in the key indicates phasing/imputation functions.
