# Supplementary material for: Comparison and assessment of family- and population-based genotype imputation methods in large pedigrees
Source: Genome Res. 2019 Jan;29(1):125–34. doi: 10.1101/gr.236315.118 (PMC6314157; doi:10.1101/gr.236315.118)
Supplement: Supplemental Material [file supp_gr.236315.118_Supplemental_Fig_S3.pdf]

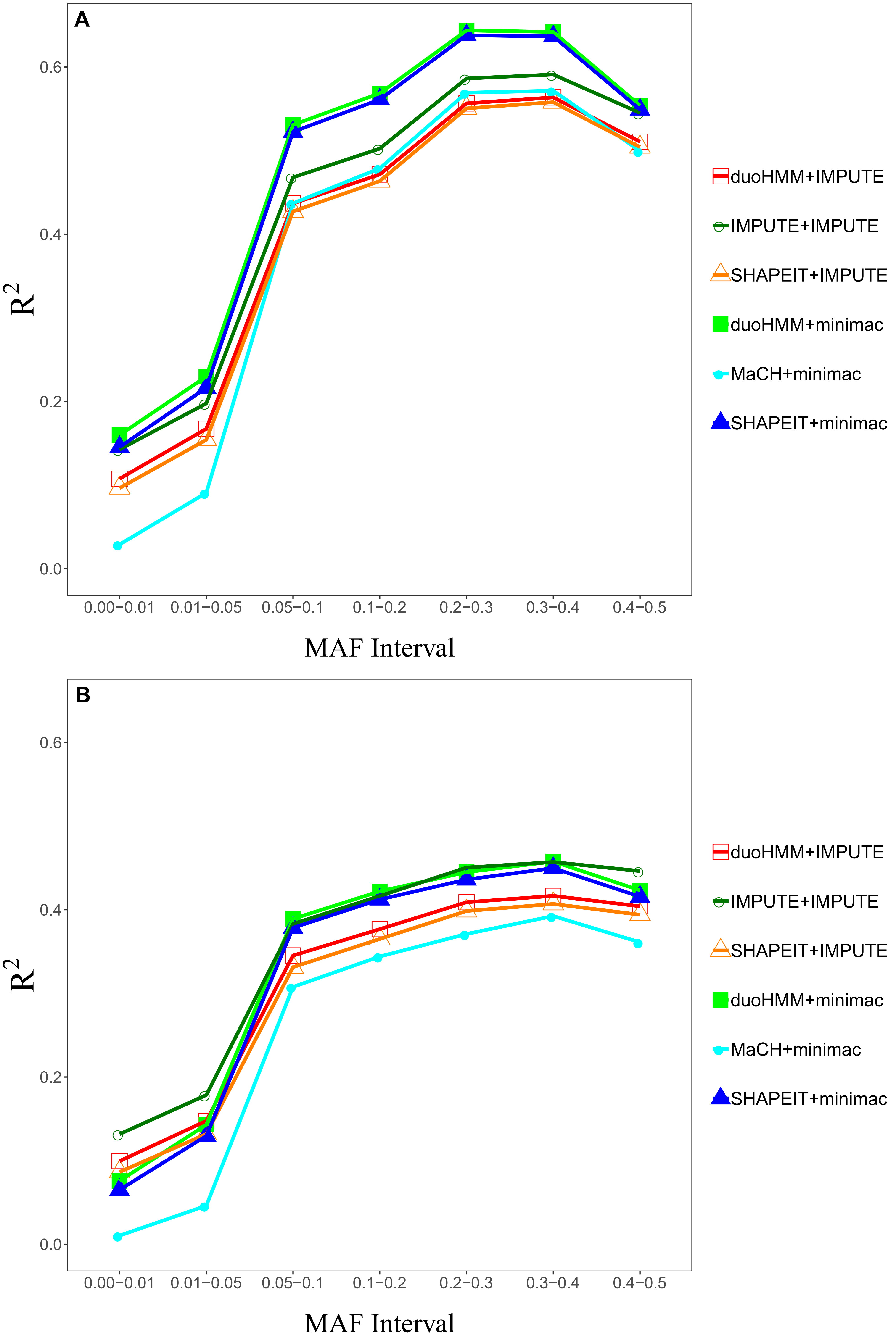

Supplemental Figure S3: Mean correlation R^2 and IQS between true and imputed genotypes for duoHMM+IMPUTE, IMPUTE+IMPUTE, SHAPEIT+IMPUTE, duoHMM+minimac, MaCH+minimac, and SHAPEIT+minimac: (A) R2 for EUR and (B) R^2 for AFR, using the random selection strategy.The first/second of a pair of programs in the key indicates phasing/imputation functions. Computation of the mean of R2 and IQS is based on a second randomly generated set of all 100 genetic datasets with a sample size of 960 subjects, each having 7,954 SNPs for EUR and 10,891 SNPs for AFR.
