# Supplementary material for: Comparison and assessment of family- and population-based genotype imputation methods in large pedigrees
Source: Genome Res. 2019 Jan;29(1):125–34. doi: 10.1101/gr.236315.118 (PMC6314157; doi:10.1101/gr.236315.118)
Supplement: Supplemental Material [file supp_gr.236315.118_Supplemental_Fig_S4.pdf]

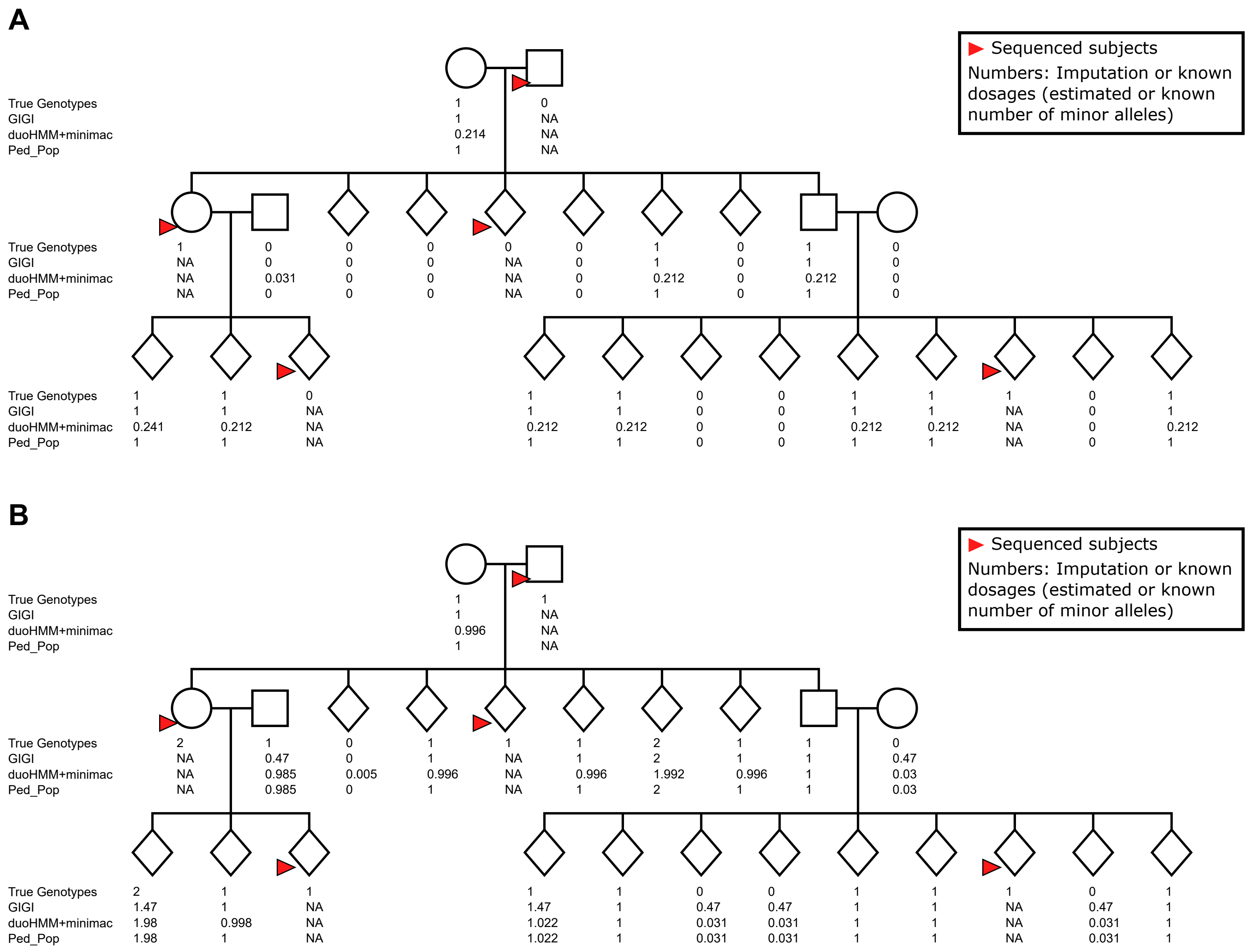

Supplemental Figure S4: GIGI, duoHMM+minimac, and Ped_Pop performance for imputing two SNPs: A) MAF=0.006 B) MAF=0.46. The pedigree size is 24 subjects. Five subjects represented by red triangles were fully observed (no missing genotypes). The numbers are the known or estimated numbers of minor alleles, i.e. dosages. The known genotypes/dosages are in the first row. GIGI's dosaes are in the second row. duoHMM+minimac's dosages are in the third row. Ped_Pop's dosages are in the fourth row.
