# Supplementary material for: Comparison and assessment of family- and population-based genotype imputation methods in large pedigrees
Source: Genome Res. 2019 Jan;29(1):125–34. doi: 10.1101/gr.236315.118 (PMC6314157; doi:10.1101/gr.236315.118)
Supplement: Supplemental Material [file supp_gr.236315.118_Supplemental_Fig_S5.pdf]

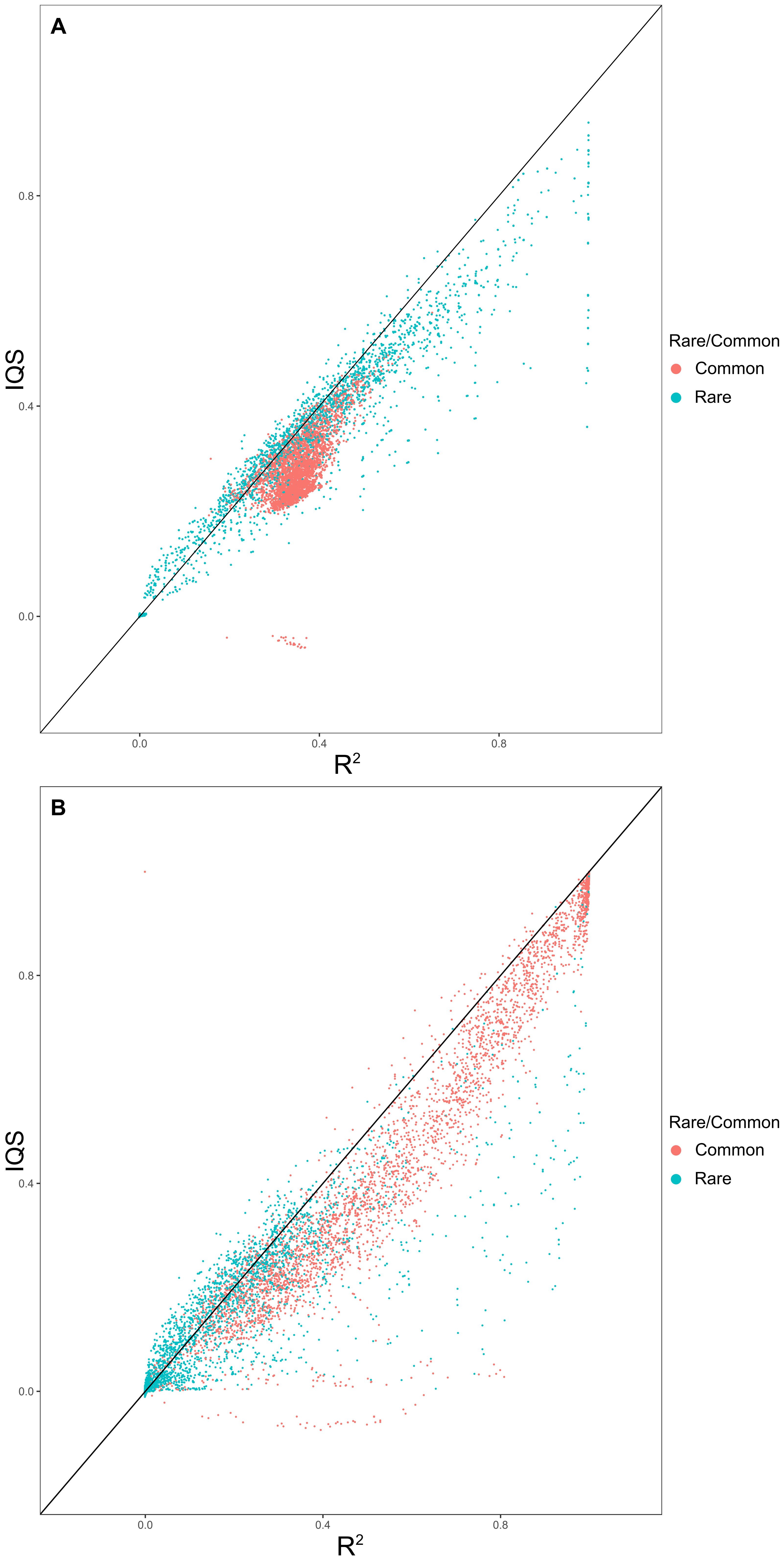

Supplemental Figure S5: Mean correlation R^2 vs IQS for (A) GIGI and (B) duoHMM+minimac, in EUR, using the Random selection strategy.
