# Supplementary material for: Comparison and assessment of family- and population-based genotype imputation methods in large pedigrees
Source: Genome Res. 2019 Jan;29(1):125–34. doi: 10.1101/gr.236315.118 (PMC6314157; doi:10.1101/gr.236315.118)
Supplement: Supplemental Material [file supp_gr.236315.118_Supplemental_Fig_S6.pdf]

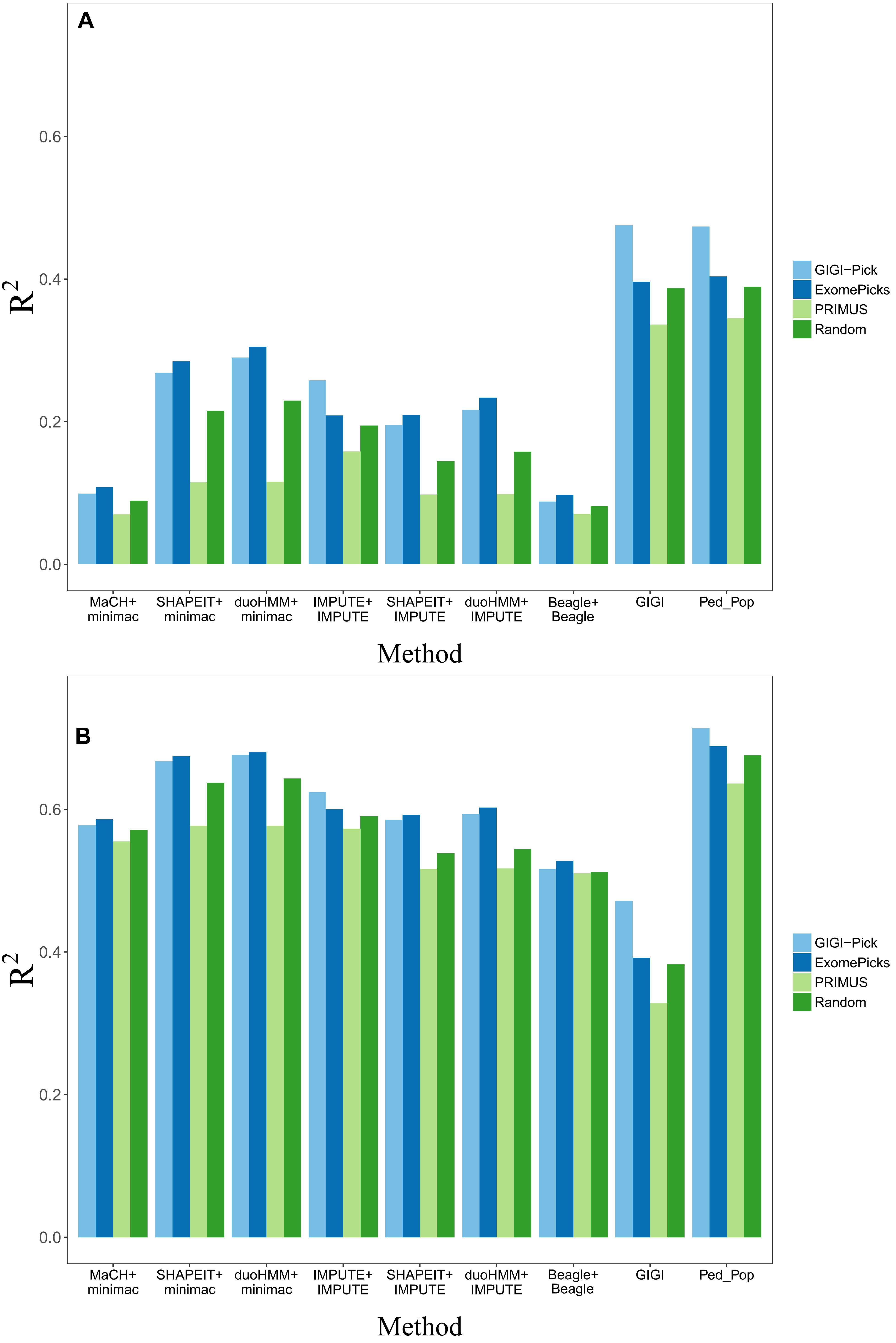

Supplemental Figure S6: Mean R^2 between true and imputed genotypes for the four selection strategies (GIGI-Pick, ExomePicks, PRIMUS, and Random selection) for all approaches in EUR for (A) rare variants (MAF in [0.00-0.05)) and (B) common variants (MAF in [0.05-0.5]).
