# Supplementary material for: Comparison and assessment of family- and population-based genotype imputation methods in large pedigrees
Source: Genome Res. 2019 Jan;29(1):125–34. doi: 10.1101/gr.236315.118 (PMC6314157; doi:10.1101/gr.236315.118)
Supplement: Supplemental Material [file supp_gr.236315.118_Supplemental_Fig_S7.pdf]

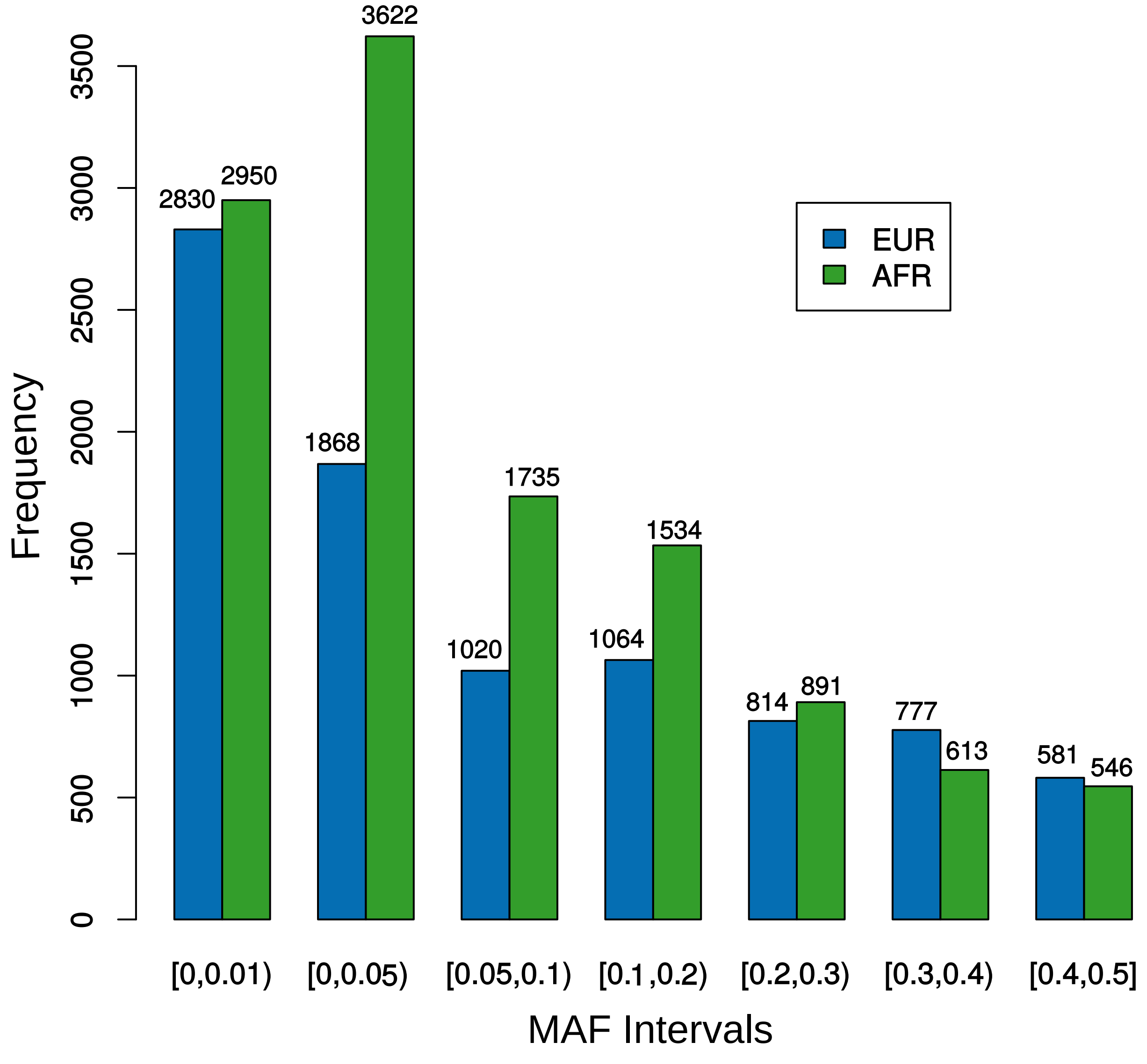

Supplemental Figure S7: MAF distribution for European and African populations in the simulated data.
