# Supplementary material for: Comparison and assessment of family- and population-based genotype imputation methods in large pedigrees
Source: Genome Res. 2019 Jan;29(1):125–34. doi: 10.1101/gr.236315.118 (PMC6314157; doi:10.1101/gr.236315.118)
Supplement: Supplemental Material [file supp_gr.236315.118_Supplemental_Methods.docx]

**Comparison and Assessment of Family- and Population-based Genotype Imputation Methods in Large Pedigrees**

Ehsan Ullah^1†^, Raghvendra Mall^1†^, Mostafa M. Abbas^1‡^, Khalid Kunji^1‡^, Alejandro Q. Nato Jr^2,3^, Halima Bensmail^1^, Ellen M. Wijsman^2,4^, Mohamad Saad^1*^

^1^Qatar Computing Research Institute, Hamad Bin Khalifa University, Doha, Qatar.

^2^Division of Medical Genetics, Department of Medicine, University of Washington, Seattle, WA, USA

^3^ Department of Biomedical Sciences, Marshall University, Huntington, WV, USA

^4^Department of Biostatistics, University of Washington, Seattle, WA, USA

^†,‡^ Contributed equally

*Corresponding author: Mohamad Saad, Qatar Computing Research Institute, Office 1117A, HBKU – Research Complex, Hamad Bin Khalifa University, P.O. Box: 5825, Doha - Qatar

Email : [msaad@hbku.edu](mailto:msaad@hbku.edu).qa; Phone : +974 4454 7746

**Running title**: Genotype Imputation in Large Pedigrees

**Keywords**: Genotype imputation, Pedigree data, Association analysis, MaCH, Minimac, SHAPEIT, EAGLE, BEAGLE, IMPUTE, GIGI, MERLIN, Imputation accuracy.

**Material and Methods**

**Family-based design imputation**

1. **Phasing Approaches**

**gl_auto implemented in MORGAN:** gl_auto (Thompson 2011) samples inheritance vectors (IVs) from the pedigree and genotype data of a set of the most informative sparse markers, usually 0.5 centiMorgan (cM) far apart. IVs represent the flow of founder alleles through pedigrees (Lander and Green 1987; Lange and Sobel 1991; Kruglyak et al. 1996; Thompson 2011) and are usually used internally for linkage analysis using the Lange-Sobel estimator (Lange and Sobel 1991). gl_auto generates IVs for each individual by using a combination of exact and Markov Chain Monte Carlo (MCMC) based estimations. In our study, IVs were sampled for each sparse marker using the following parameters: 2,500 MCMC burn-in iterations, sampling by scan, and 50,000 MCMC iterations with progress checked every 20,000 iterations (L-sampler = 0.2), saving 1000 realizations. Use of gl_auto allows multipoint IBD estimation on the complete pedigree, which is comparable to the pre-phasing step of population-based imputation software.

1. **Imputation Approaches**

**GIGI:** GIGI (Cheung et al. 2013) is a pedigree-based imputation computer program. It uses a sparse set of “framework markers” typed on most subjects plus a set of “dense markers” typed on a few subjects. The imputation relies on correlation resulting from inheritance in pedigrees through the inheritance of shared segments of a chromosome as represented by IVs (Thompson 2011). In brief, this imputation approach consists of four steps: (1) sample IVs at the positions of framework markers, conditional on the observed genotypes at these markers using MCMC sampling in large pedigrees or the exact conditional distribution in small pedigrees, (2) sample IVs at the positions of dense markers conditional on the pedigree structure and IVs sampled at the positions of the framework markers and the meiotic map, (3) estimate the probability distribution for each unobserved genotype at the dense marker positions conditional on all observed dense marker genotypes, their allele frequencies and position-specific IVs corresponding to the dense markers, and (4) call genotypes using the estimated probabilities and user-specified thresholds.

**MERLIN:** MERLIN (Abecasis et al. 2002) is a pedigree-based program used for many types of analysis including linkage analysis and genotype imputation (Burdick et al. 2006). For imputation analysis, MERLIN relies on the Lander-Green (Lander and Green 1987) algorithm for transversing the pedigree, which scales linearly in the number of markers, but exponentially in the number of subjects, therefore requiring too much memory to run on most pedigrees because of their large size. To get results from MERLIN, we had to split pedigrees into smaller sub-pedigrees. Because of the computational burden, we ran MERLIN only for the EUR simulation. Nonetheless, the results from the EUR simulation are generalizable to the AFR simulation because the same simulated IBD-structure was used in both ancestries.

**Splitting pedigrees for MERLIN:** The amount of memory to run MERLIN can be predicted by the number of bits in the pedigree (Kruglyak et al. 1996), $bits=2n-f,$ where $n$ is the number of non-founders and $f$ is the number of founders. The pedigrees we are using ranged from 5 to 165 bits. Classifying pedigrees into small and large can be approximately determined by the number of bits. In our data, imputation of pedigrees with 19 bits required 110 Gb of memory to impute 8,954 SNPs. Therefore, we decided to split large pedigrees into small computable sub-pedigrees defined as those a maximum of 19 bits. Automated methods for subdividing the pedigree structures exist, such as PedCut (Liu et al. 2008) and PedStr (Kirichenko et al. 2009). However, we found them unsatisfactory, resulting in excessively small sub-pedigrees without the flexibility to ensure that at least some sequenced subjects are in each sub-pedigree. We instead opted to manually construct the sub-pedigrees to include a greater number of subjects in each sub-pedigree and to be close to the upper limit of 19 bits, while including both vertical and horizontal relationships (Grand-parents, Parents, Offspring, Siblings, etc.). In order to have close to 19 bits, we often included the same individuals in several of the sub-pedigrees. In these cases, we retained the imputation results for these individuals from the largest sub-pedigree when combining the results.

**Population-based design imputation**

1. **Phasing Approaches**

**SHAPEIT (v2):** SHAPEIT (Delaneau et al. 2012) is a hidden Markov model-based (HMM) approach in which the haplotypes of each sample are updated iteratively and inference is done using Gibbs sampling. The haplotypes of a sample are updated conditionally depending upon the current haplotype estimates of all other samples. SHAPEIT introduces approximations to improve performance. The first approximation includes creation of a graph capturing the haplotype structure using a greedy approach to collapse the haplotypes being conditioned on. The main advantage of this approximation is the use of all haplotypes, relative to other methods that use only a subset of haplotypes. The second approximation involves using a different space to represent haplotypes that are consistent with a subject’s genotypes. SHAPEIT also incorporates a surrogate family phasing approach, which caters for extended sharing of segments between subjects.

**DuoHMM:** DuoHMM (O'Connell et al. 2014) is an HMM based approach that combines the results of SHAPEIT with the given pedigree information for inheritance pattern inference of each meiosis at all sites of all chromosomes. DuoHMM corrects phasing errors that are inconsistent with pedigree information. Inclusion of pedigree information offers certain advantages such as correction of switch errors, detection of recombination events and genotyping errors. The HMM in duoHMM uses a constant number of hidden states, four in this case, and sixteen possible transitions between states per meiosis. DuoHMM scales linearly with the number of non-founders and the number of SNPs. For *n* number of non-founders and *L* number of SNPs, the complexity of the duoHMM algorithm is O(*nL*).

**IMPUTE (v2):** IMPUTE (Howie et al. 2009) estimates haplotypes using Markov Chain Monte Carlo (MCMC) algorithms. IMPUTE probabilistically samples phased haplotypes for each subject, conditional on the current haplotype guesses for the rest of the subjects. IMPUTE uses k templates from the set of available haplotypes of a subject by computing the Hamming distance between the current haplotype and the set of available haplotypes to reduce the runtime. IMPUTE also incorporates surrogate family phasing approach, which incorporates extended sharing of segments between subjects to improve the phasing process.

**MaCH:** MaCH (Li et al. 2010) uses a Markov Chain haplotyping algorithm. To estimate the haplotypes, MaCH starts by randomly generating a pair of haplotypes that is compatible with available genotypes for each sampled subject. These initial haplotypes are further refined by performing a number of iterations. During each iteration, a new pair of haplotypes is sampled for each subject using an HMM, which describes the haplotype pair as an imperfect mosaic of the other haplotypes. After several iterations (typically 20-100), a consensus haplotype can be constructed by merging the haplotypes sampled from each round. The computational complexity of the MaCH algorithm is O(states^2^ × rounds) and increases quadratically with the number of states. MaCH was recently used for genotype phasing and forms a pre-phasing step that is followed by Minimac for imputation (Howie et al. 2012; Fuchsberger et al. 2015).

**BEAGLE (v4.1):** BEAGLE (Browning and Browning 2007) is based on a localized haplotype clustering model, which is an empirical LD model that adapts to the local structure of the data by modeling haplotype frequencies on a local scale. The localized haplotype clustering model initially clusters the haplotypes at each marker such that the haplotypes in the same cluster tend to have similar probabilities for alleles at downstream markers (Browning and Browning 2011). Then, it defines a diploid HMM with ordered pairs of edges at each level of the model to find the most likely haplotype pairs for each subject given the subject's known genotypes.

**EAGLE (v2):** EAGLE (Loh et al. 2016) estimates haplotype phase using the BEAGLE algorithm (Browning and Browning 2011) either within a genotyped cohort or using a phased reference panel. EAGLE is now the default phasing method used by the Sanger and Michigan imputation servers and uses a new very fast HMM-based algorithm that improves speed and accuracy over existing methods via two key ideas: a new data structure based on the positional Burrows-Wheeler transform (Durbin 2014) and a rapid search algorithm that explores only the most relevant paths through the HMM. The algorithm in EAGLE (v2) provides similar speed compared to EAGLE (v1) but with much greater accuracy at sample sizes <50,000 subjects.

1. **Imputation Approaches**

**IMPUTE (v2):** For genotype imputation, IMPUTE searches for reference haplotypes that share high sequence identity with the haplotypes of the subject being imputed. IMPUTE considers the genetic distance of a locus of interest to its neighbors for imputation and assumes a uniform mutation rate across the whole genome.

**Minimac (v3):** Minimac (Fuchsberger et al. 2015) requires the haplotypes for all subjects, which can be obtained by using one of the phasing methods described above. Minimac then imputes missing genotypes using these estimated haplotypes and the haplotypes of the reference dataset. Similar to IMPUTE, Minimac searches for reference haplotypes that share high sequence identity with the haplotypes of the subject being imputed. Recently, a multiprocessor version of Minimac was developed, namely minimac2-omp (Fuchsberger et al. 2015), which uses the OpenMP protocol to support multi-threading for a much larger throughput. It also allows for handling very large reference panels with hundreds or thousands of haplotypes.

**BEAGLE (v4.1)**: BEAGLE (Browning and Browning 2016) considers a fixed genetic distance, 0.005 cM by default, to combine the sets of consecutive markers within that distance into an aggregate set of genotyped markers in the study dataset. BEAGLE uses the set of all ordered pairs such that the first element is an aggregate genotyped marker and the second element is a reference haplotype, to build an HMM. Restricting the calculations to genotyped markers in the target samples and their neighborehood reduces the running time and memory consumption for BEAGLE. BEAGLE was designed to work on multicore systems using multiple threads.

**Ped_Pop:** Ped_pop (Saad and Wijsman 2014) combines family- and population-based imputation results to benefit from both IBD and LD information. In fact, family-based imputation relies on IBD and population-based imputation relies on LD. The weakness of family-based imputation that it suffers for common variant imputation and the weakness of population-based imputation that it suffers for rare variant imputation. The idea of Ped_Pop is to compare the variance of the three genotype posterior probabilities between one family-based and one population-based method, and select the probabilities with the highest variance. In our study, we combined the family-based imputation results of GIGI and the combination of duoHMM and Minimac for population-based imputation. The rational of this combination is that for family-based imputation, GIGI performed best for both EUR and AFR populations. For population-based imputation, the combination of duoHMM and Minimac performed best in the EUR population and second best (but very close to the combination of IMPUTE and IMPUTE) for phasing and imputation in the AFR population.

**Selection of Reference Dataset**

Random: In the random selection strategy, 20% of subjects of each pedigree were randomly selected. The selection was done 100 times, once for each of the 100 simulated genotype datasets to cover a large variety of selection combinations.

GIGI-Pick: GIGI-Pick (Cheung et al. 2014) selects the most informative individuals for sequencing by balancing the selection of closely related individuals within the pedigree against the selection of more distant relatives to help in the phasing of genotypes and observing unique founder alleles respectively. GIGI-Pick defines a metric called coverage, which is the expected percentage of alleles that can be imputed for a random variant. Coverage=0 occurs if no alleles can be imputed (as may happen if no sequenced subjects are available), while coverage=1 occurs if all alleles can be imputed. GIGI-Pick selects the set of subjects that maximizes the coverage.

ExomePicks**:** ExomePicks selects subjects for sequencing using multiple generations (from the oldest to youngest generations). ExomePicks identifies the haplotypes across loci by selecting every founder and at least one offspring per founder. Selecting every founder will identify all segregating chromosomes while selecting the offspring will help to determine the marker phase.

PRIMUS: PRIMUS (Staples et al. 2013) is primarily a tool that uses estimated kinship measurements of a set of subjects to reconstruct pedigrees. PRIMUS also identifies a set of maximal unrelated subjects, which can be used for subject selection (Uricchio et al. 2012). The idea behind PRIMUS for subject selection is that selecting a set of maximal unrelated subjects may cover most of the available genetic diversity. However, this approach suffers from the absence of related subjects who help with genotype phasing, which is a crucial factor for imputation performance.

**REFERENCES**

Abecasis GR, Cherny SS, Cookson WO, Cardon LR. 2002. Merlin-rapid analysis of dense genetic maps using sparse gene flow trees. *Nature Genetics* **30**: 97-101.

Browning BL, Browning SR. 2016. Genotype Imputation with Millions of Reference Samples. *Am J Hum Genet* **98**: 116-126.

Browning SR, Browning BL. 2007. Rapid and accurate haplotype phasing and missing-data inference for whole-genome association studies by use of localized haplotype clustering. *American Journal of Human Genetics* **81**: 1084-1097.

Browning SR, Browning BL. 2011. Haplotype phasing: existing methods and new developments. *Nature Reviews Genetics* **12**: 703-714.

Burdick JT, Chen WM, Abecasis GR, Cheung VG. 2006. In silico method for inferring genotypes in pedigrees. *Nature Genetics* **38**: 1002-1004.

Cheung CYK, Blue EM, Wijsman EM. 2014. A statistical framework to guide sequencing choices in pedigress. *American Journal of Human Genetics* **94**: 257-267.

Cheung CYK, Thompson EA, Wijsman EM. 2013. GIGI: An approach to effective imputation of dense genotypes on large pedigrees. *American Journal of Human Genetics* **92**: 504-516.

Delaneau O, Marchini J, Zagury JF. 2012. A linear complexity phasing method for thousands of genomes. *Nature Methods* **9**: 179-181.

Fuchsberger C, Abecasis GR, Hinds DA. 2015. minimac2: faster genotype imputation. *Bioinformatics* **31**: 782-784.

Howie B, Fuchsberger C, Stephens M, Marchini J, Abecasis GR. 2012. Fast and accurate genotype imputation in genome-wide association studies through pre-phasing. *Nat Genet* **44**: 955-959.

Howie BN, Donnelly P, Marchini J. 2009. A flexible and accurate genotype imputation method for the next generation of genome-wide association studies. *PLoS Genet* **5**: e1000529.

Kirichenko AV, Belonogova NM, Aulchenko YS, Axenovich TI. 2009. PedStr software for cutting large pedigrees for haplotyping, IBD computation and multipoint linkage analysis. *Ann Hum Genet* **73**: 527-531.

Kruglyak L, Daly MJ, Reeve-Daly MP, Lander ES. 1996. Parametric and nonparametric linkage analysis: a unified multipoint approach. *Am J Hum Genet* **58**: 1347-1363.

Lander ES, Green P. 1987. Construction of multilocus genetic linkage maps in humans. *Proc Natl Acad Sci U S A* **84**: 2363-2367.

Lange K, Sobel E. 1991. A random walk method for computing genetic location scores. *Am J Hum Genet* **49**: 1320-1334.

Li Y, Willer CJ, Ding J, Scheet P, Abecasis GR. 2010. MaCH: using sequence and genotype data to estimate haplotypes and unobserved genotypes. *Genet Epidemiol* **34**: 816-834.

Liu F, Kirichenko A, Axenovich TI, van Duijn CM, Aulchenko YS. 2008. An approach for cutting large and complex pedigrees for linkage analysis. *European Journal of Human Genetics* **16**: 854-860.

Loh PR, Danecek P, Palamara PF, Fuchsberger C, Y AR, H KF, Schoenherr S, Forer L, McCarthy S, Abecasis GR et al. 2016. Reference-based phasing using the Haplotype Reference Consortium panel. *Nat Genet* **48**: 1443-1448.

O'Connell J, Gurdasani D, Delaneau O, Pirastu N, Ulivi S, Cocca M, Traglia M, Huang J, Huffman JE, Rudan I et al. 2014. A general approach for haplotype phasing across the full spectrum of relatedness. *PLoS Genet* **10**: e1004234.

Saad M, Wijsman EM. 2014. Combining family- and population-based imputation data for association analysis of rare and common variants in large pedigrees. *Genet Epidemiol* **38**: 579-590.

Staples J, Nickerson DA, Below JE. 2013. Utilizing graph theory to select the largest set of unrelated individuals for genetic analysis. *Genet Epidemiol* **37**: 136-141.

Thompson EA. 2011. The structure of genetic linkage data: from LIPED to 1M SNPs. *Human Heredity* **71**: 86-96.

Uricchio LH, Chong JX, Ross KD, Ober C, Nicolae DL. 2012. Accurate imputation of rare and common variants in a founder population from a small number of sequenced individuals. *Genet Epidemiol* **36**: 312-319.
